# Structures of *Cutibacterium acnes* hyaluronate lyases suggest a correlation between active site opened/closed state and conformation of abutting loop

**DOI:** 10.17912/micropub.biology.001237

**Published:** 2024-07-30

**Authors:** Randall McNally, Ramachandran Murali

**Affiliations:** 1 Department of Biomedical Sciences, Research Division of Immunology, Cedars-Sinai Medical Center, Los Angeles, California, United States

## Abstract

The structures of hyaluronate lyases from two *Cutibacterium acnes* strains have been reported recently and show open catalytic clefts. We compared these open structures with more closed structures of homologous lyases and found that the conformation of a loop that abuts the catalytic cleft is seemingly correlated with the opening and closing of the cleft. We illustrate that the loop conformation seen in the open lyase appears incompatible with a closed catalytic cleft, and vice versa; however, mutations designed to disrupt the loop conformation did not significantly affect catalytic activity.

**Figure 1. Structural features of helix α2, helix α13, and the α12-α13 loop:**
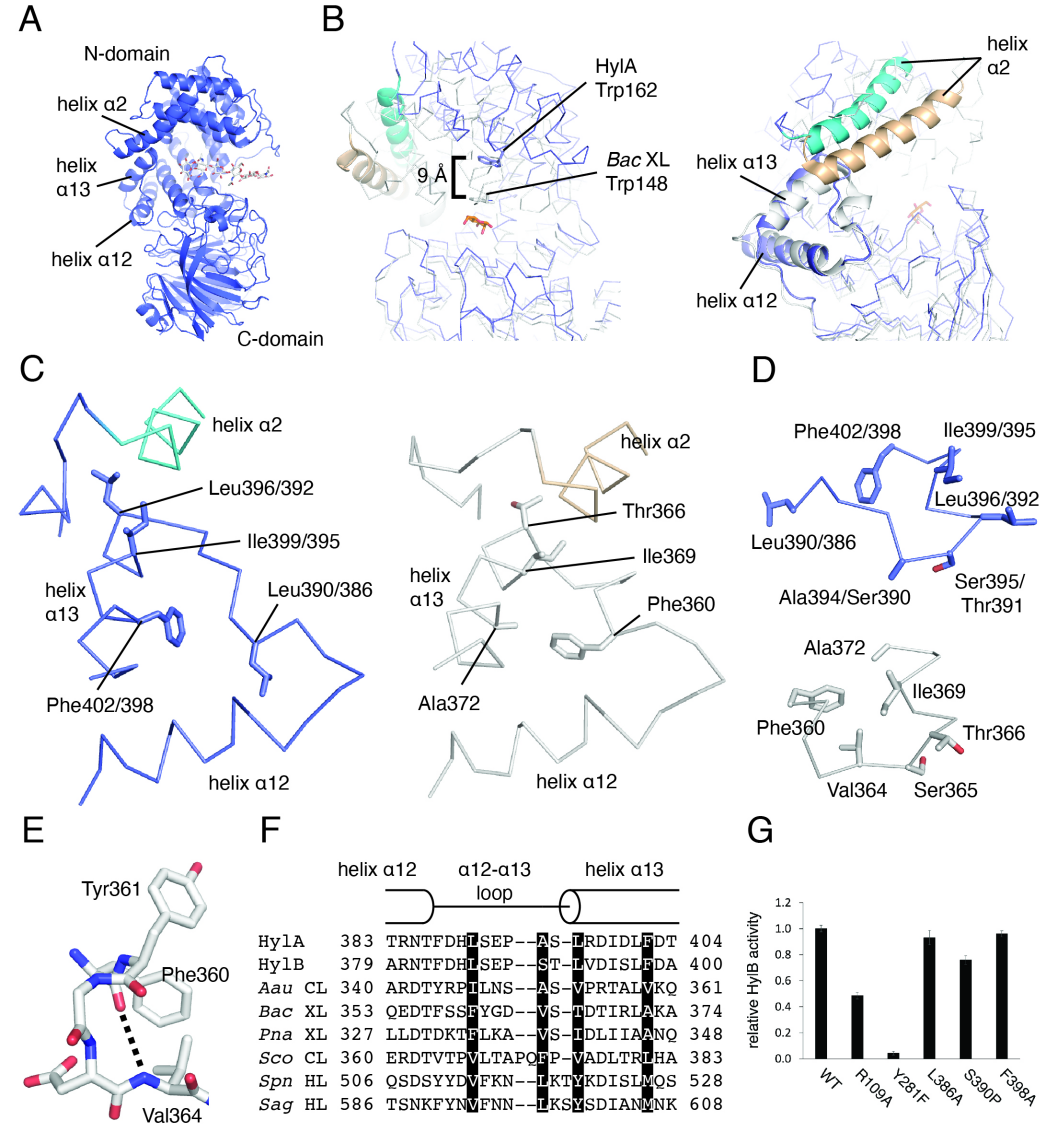
A) Structure of HylA (PDB 8FYG) (Hajam et al., 2023) with hexasaccharide hyaluronan (white) modeled in catalytic cleft based on overlay of HylA with *S. agalactiae* hyaluronate lyase in complex with hyaluronan (PDB 1LXM) (Mello et al., 2002). B) Overlay of HylA and *Bac* XL in complex with mannose (PDB 2E22) (Maruyama et al., 2007), aligned on their C-domains. HylA is colored blue; *Bac* XL is white. Helix α2 is colored cyan and tan for HylA and *Bac* XL, respectively. Mannose is colored orange. C) Helix α2, α12-α13 loop, and helix α13 of HylA (left panel) and *Bac* XL (right panel). Residue numbers on left panel correspond to those for HylA followed by HylB. D) The α12-α13 loop of HylA (top panel) and *Bac* XL (bottom panel). Residue numbers on top panel correspond to those for HylA followed by HylB. E) Helical interaction within the α12-α13 loop of *Bac* XL. Dotted line represents a hydrogen bond that forms an α-helical interaction. F) Sequence alignment of helix α12, α12-α13 loop, and helix α13 of structurally known gram-positive GAG lyases. Sequences were aligned according to crystal structures. Key residues of proposed opening/closing mechanism are highlighted. *Aau* CL, *A. aurescens* chondroitin AC lyase; *Bac* XL, *Bacillus* sp. strain GL1 xanthan lyase; *Pna* XL, *P. nanensis* xanthan lyase; *Sco* CL, *S. coelicolor* chondroitin AC lyase; *Spn* HL, *S. pneumoniae* hyaluronate lyase; *Sag* HL, *S. agalactiae* hyaluronate lyase. G) Hyaluronate lyase activity of HylB and mutants. HylB at concentration 15 ng/mL and high molecular weight hyaluronic acid at concentration 0.2 mg/mL were monitored at wavelength 232 nm. Reaction volume was 100 μL. Assay buffer contained 100 mM Na acetate pH 5.5, 10 mM CaCl _2_ , and 0.5 mM TCEP. Results are scaled relative to wild-type HylB. Error bars are standard error of three measurements.

## Description

Glycosaminoglycan (GAG) lyases secreted by bacteria degrade polysaccharide GAGs within the extracellular matrix for host cell invasion (Jedrzejas, 2000) , concomitantly eliciting inflammatory responses. GAG lyases act on the glycosidic linkage of substrates such as hyaluronic acid, chondroitin sulfate, xanthan, and heparin through a β-elimination mechanism and feature a conserved three-dimensional architecture that consists of a mostly α-helical N-terminal domain, a C-terminal domain comprising mainly of β-strands, and a catalytic site in a cleft largely within the N-domain (Stern & Jedrzejas, 2006) .

The gram-positive *Cutibacterium acnes* colonizes the skin of subjects with acne and healthy skin alike. However, genetic analysis has shown that strains of *C. acnes* can be categorized by their association with acneic or healthy skin, and that the variant of hyaluronate lyase expressed by a strain correlates with its hosts’ propensity to develop acne (Fitz-Gibbon et al., 2013; McLaughlin et al., 2019) . HylA is the representative hyaluronate lyase from strains of *C. acnes* associated with acneic skin; HylB is expressed by strains associated with healthy skin. In a previous study, we reported the X-ray crystal structures of HylA and HylB, both solved without substrate (Hajam et al., 2023) . Befitting enzymes with 74% identity between them, the structures are highly similar; HylA and HylB overlay with a r.m.s.d. of 0.8 Å over 751 residues.

A survey of the Protein Data Bank reveals that, in addition to HylA and HylB, structures for six GAG lyases from gram-positive bacteria have been deposited: xanthan lyases from *Bacillus* sp. strain GL1 and *Paenibacillus* *nanensis* , chondroitin AC lyases from *Streptomyces coelicolor* and *Arthrobacter aurescens* , and hyaluronate lyases from *Streptococcus* *agalactiae* and *Streptococcus pneumoniae* (Li et al., 2000; Li & Jedrzejas, 2001; Hashimoto et al., 2003; Lunin et al., 2004; Elmabrouk et al., 2011; Jensen et al., 2019) . Each of these enzymes are homologous to HylA and HylB, with sequence identities ranging from 23-37% vs. HylA, and are structurally homologous as well, with root mean square deviations vs. HylA ranging from 2.2-3.4 Å.

Comparison of the structures of HylA/B to those of the six other GAG lyases from gram-positive bacteria reveals that the substrate binding clefts in the structures of HylA/B are more open than all. This is exemplified by the open position of helix α2 (helix numbering follows the convention established by Li et al., 2000), which forms part of the “lid” that closes over the active site (Figures 1A and 1B), and the position of HylA Trp162, which is approximately 9 Å from the position of the corresponding Trp in *Bacillus* sp. strain GL1 xanthan lyase ( *Bac* XL), a residue that interacts with substrate (Hashimoto et al., 2003; Maruyama et al., 2005, 2007) ( Figure 1B ).

Gram-positive GAG lyases, including HylA/B, feature a triangularly arranged element comprised of helix α12, helix α13, and the α12-α13 loop that connects them (Figures 1A and 1B) abutting the catalytic cleft. The side chain of the N-terminal residue of helix α13 acts as a “pin” inserted into a pocket adjacent to helix α2 ( Figure 1C ). We observed that the conformation of the 10-residue α12-α13 loop in HylA/B is different from that of all other structures of gram-positive GAG lyases.

To demonstrate how the α12-α13 loop of HylA/B is different, we compare the α12-α13 loops of the open HylA/B with that of the closed *Bac* XL, which has the same length of loop as HylA/B. We can establish that the *Bac* XL structure is closed because it is complexed with a product analog, and residues of the catalytic triad are in orientation for catalysis (Hashimoto et al., 2003; Maruyama et al., 2005, 2007) . The comparison shows that the conformation of the α12-α13 loop in HylA/B is incompatible with the closed form of the enzyme. This is illustrated by a “domino effect” that can be traced beginning with the movement of loop residue *Bac* XL Phe360 and culminating with the closing of the “lid.” *Bac* XL Phe360 is flipped away relative to its equivalent residue in HylA/B (Leu390/386) toward *Bac* XL Ala372 on helix α13 ( Figure 1C ). Steric clash between *Bac* XL Phe360 and *Bac* XL Ala372 then forces a rotation of helix α13 ( Figure 1C ), which necessitates a rearrangement of the α12-α13 loop: *Bac* XL Val364 is flipped from the solvent-facing position seen in its equivalent in HylA/B (Ala394/Ser390) towards the pocket formed by *Bac* XL Phe360 and *Bac* XL Ala372 and forms an α-helical interaction with *Bac* XL Phe360 (Figures 1D and 1E). The combined rearrangement (rotation of helix α13, reorganization of α12-α13 loop) pulls the “pin” residue *Bac* XL Thr366 (equivalent to HylA/B Leu396/392), and helix α2 along with it, towards the front of the substrate binding pocket, concurrent with the closing of the “lid” ( Figure 1C ).

We thus define the closed conformation of the α12-α13 loop as follows, as seen in *Bac* XL: 1. *Bac* XL Phe360 is oriented to pack against *Bac* XL Ala372; 2. *Bac* XL Val364 is flipped towards *Bac* XL Phe360 and forms an α-helical or 3 _10_ -helical interaction with a preceding residue (Figures 1C-E). In the open conformation, the following applies, as seen in HylA/B: 1. Leu390/386 is flipped away from Phe402/398; 2. Ala394/Ser390 is solvent-exposed, obviating the helical interaction (Figures 1C and 1D). Sequence alignment of the α12-α13 loop and adjacent residues for gram-positive GAG lyases reveals that while there is little conservation in this region, conservation of hydrophobic character at the positions analogous to HylA/B Leu390/386 and Phe402/398 supports such a role for these residues ( Figure 1F ).

Because of the steric considerations described in this mechanism, the three structural features connected by the “pin” residue (helix α13, the α12-α13 loop, and helix α2) must move in concert; helix α2 cannot be in the closed “pulled forward” position if the loop and helix α13 (and thus the “pin”) are in the open position, and vice versa. This forced compatibility between these features potentially underscores an intrinsic correlation between cleft openness and the α12-α13 loop conformation.

That the α12-α13 loop apparently changes conformation with the opening and closing of the catalytic site led us to ask whether the loop is integral to the enzyme’s opening and closing mechanism, and thus, its function. We therefore mutated three residues in HylB identified as crucial to the conformational changes associated with the α12-α13 loop. HylB L386A and F398A (equivalent to *Bac* XL Phe360 and Ala372, respectively) were designed to disrupt the movement of helix α13 that results from the flipping of Leu386 by reducing steric forces. Next, HylB S390P (analogous to *Bac* XL Val364) was designed to disrupt the helical interaction by eliminating the proton donor of the hydrogen bond.

To test the effects of these mutations, we used a spectrophotometric assay that monitors hyaluronic acid cleavage by measuring formation of the double bond between C4 and C5 of the glucuronic acid moiety during the β-elimination reaction catalyzed by the enzyme (Li et al., 2000) . We found that HylB L386A and F398A had no effect on enzyme activity relative to wild-type HylB, and that the effect of S390P was a modest reduction of approximately 20% ( Figure 1G ). The control mutation of a predicted hyaluronic acid-binding residue, HylB R109A, resulted in a 50% loss in activity, while mutation of the tyrosine of the catalytic triad (HylB Y281F) resulted in near-total loss of activity ( Figure 1G ). Thus, this experiment does not establish that the loop plays a significant role in controlling the catalytic rate of the enzyme.

Why structural examination evinces coupling between the α12-α13 loop and the catalytic cleft while our experimental mutations do not is unclear. It is possible that individual mutations of HylB Leu386 and Phe398 to the smaller alanine may not have been severe enough to relieve the steric impingement these residues are proposed to exert upon one another; mutating both residues together to alanine may thus show greater effect. A further complication is that even if these mutations were successful in eliminating the steric impingement, both the opened and closed conformations of the loop would still be available. Likewise, even if the S390P mutant is deficient in stabilizing the closed conformation of the loop, the closed conformation would still be sterically possible. This could limit the effectiveness of the mutations in preventing the enzyme from opening and closing in response to substrate binding and release.

In conclusion, we propose that the body of crystal structures of gram-positive GAG lyases to date suggests a correlation between the disposition of the α12-α13 loop and the open/closed state of these enzymes. However, this correlation remains to be confirmed by structures of HylA/B with substrate bound or of other gram-positive GAG lyases in the fully open conformation. Structure prediction tools such as AlphaFold may also prove useful for exploring the conformational possibilities of lyases for which no structure has been determined. Further, it is unclear whether the loop conformation plays an integral part in the opening/closing action. Thus, more comprehensive studies that examine the role of the α12-α13 loop in catalytic activity, cleft opening/closing dynamics, and substrate binding and release are necessary to determine the importance of this motif on enzyme function. A detailed understanding of the loop in the opened and closed configurations may be critical for the development of therapeutics for acne treatment.

## Methods

*Protein expression and purification.* HylB and mutants were expressed and purified as described previously (Hajam et al., 2023) .

*Sequence and structure comparisons* . Structural overlays were generated using PyMOL (The PyMOL Molecular Graphics System, Version 2.0 Schrödinger, LLC). Calculation of sequence identities between GAG lyases and root mean square deviations between crystal structures of GAG lyases were performed using the Dali Server (Holm & Laakso, 2016) .

*Hyaluronic acid cleavage spectrophotometric assay.* HylB at concentration 15 ng/mL and high molecular weight hyaluronic acid (HMW-HA) at concentration 0.2 mg/mL were added to a 96-well UV-Star clear microplate (Greiner Bio-One, #655801). Reactions were monitored over 10 min at wavelength 232 nm using an Infinite M200 Pro UV spectrophotometer (Tecan). Reaction volume was 100 μL. Assay buffer contained 100 mM Na acetate pH 5.5, 10 mM CaCl _2_ , and 0.5 mM TCEP. Reaction velocities (absorbance units/sec) were obtained using the slope calculated by Magellan software v. 7.0 over reaction time 1-9.5 minutes. All reactions were performed in triplicate. HMW-HA was hyaluronic acid sodium salt from rooster comb, Sigma #H5388, MW 1-4 million Da. This method was adapted from a previous work (Jedrzejas et al., 1998) .
